# Contextual diversity and anchoring: Null effects on learning word forms and opposing effects on learning word meanings

**DOI:** 10.1177/17470218231218990

**Published:** 2024-09-17

**Authors:** Jiayin Li, Louise Wong, Catarina Rodrigues, Rachael C Hulme, Holly Joseph, Fiona E Kyle, J S H Taylor

**Affiliations:** 1 School of Psychology and Clinical Language Sciences, University of Reading, Reading, UK; 2 Department of Language and Cognition, Division of Psychology and Language Sciences, University College London, London, UK; 3 Department of Psychology, Centre for Applied Behavioural Sciences, Heriot-Watt University, Edinburgh, Scotland, UK; 4 Institute of Education, University of Reading, Reading, UK

**Keywords:** Contextual diversity, word learning, orthography, semantics, anchoring

## Abstract

Words that appear in many contexts/topics are recognised faster than those occurring in fewer contexts. However, contextual diversity benefits are less clear in word learning studies. Mak et al. proposed that diversity benefits might be enhanced if new word meanings are anchored before introducing diversity. In our study, adults (*N* = 288) learned meanings for eight pseudowords, four experienced in six topics (high diversity) and four in one topic (low diversity). All items were first experienced five times in one topic (anchoring phase), and results were compared to Norman et al. which used a similar paradigm without an anchoring phase. An old-new decision post-test (did you learn this word?) showed null effects of contextual diversity on written form recognition accuracy and response time, mirroring Norman et al. A cloze task involved choosing which pseudoword completed a sentence. For sentences situated in a previously experienced context, accuracy was significantly higher for pseudowords learned in the low diversity condition, whereas for sentences situated in a new context, accuracy was non-significantly higher for pseudowords learned in the high diversity condition. Anchoring modulated these effects. Low diversity item accuracy was unaffected by anchoring. However, for high-diversity items, accuracy in familiar contexts was better in the current experiment (anchoring) than in Norman et al. (non-anchoring), but accuracy in new contexts did not differ between the two experiments. These results suggest that anchoring facilitates meaning use in familiar contexts, but not generalisation to new contexts, nor word recognition in isolation.

## Introduction

Reading provides a useful medium for vocabulary acquisition (Landauer & Dumais, 1997). Indeed, from mid-childhood onwards, most new words are learned through reading rather than spoken language (Nagy et al., 1987). Multiple exposures to a novel word in meaningful texts can establish a lexical representation (Hulme et al., 2019), but it is the quality of that representation that determines whether we retrieve words efficiently and reliably (Perfetti & Hart, 2002). Specifically, the lexical quality hypothesis proposes that higher-quality word representations are “more fully-specified, more stable and less context-bound” than lower-quality representations (Nation, 2017, p. 2). Building on this, the lexical legacy hypothesis suggests that the varying contexts provided by reading experience can culminate in differences in lexical quality (Nation, 2017). Each encounter with a word in a semantically informative context strengthens and enriches its representation which then impacts lexical and semantic processing in future. Combining the lexical legacy and lexical quality hypotheses suggests that experiencing a word in diverse contexts will lead to (i) a less context-bound representation, allowing it to be more easily recognised in isolation, and (ii) a more flexible representation, allowing readers to generalise to understand and use the word in new contexts.

In line with the first of these arguments, words that are experienced in more diverse contexts (such as predicament) are recognised faster in a lexical decision task, than those experienced in less diverse contexts (such as perjury), even after accounting for word frequency (Adelman et al., 2006; Hoffman & Woollams, 2015; Hsiao et al., 2020; Johns et al., 2012). However, these same studies found that high relative to low diversity items are disadvantaged in semantic tasks, such as synonym judgement (Hoffman & Woollams, 2015; Johns et al., 2012) or definition matching (Hsiao et al., 2020). This may be because multiple contexts are likely to be activated simultaneously for high-diversity words, hindering recall of the precise context or meaning required by such tasks. This idea is supported by the fact that concreteness judgement, which requires less precise meaning knowledge, often shows a high diversity advantage (e.g., Pexman et al., 2008; Yap et al., 2011). However, these studies do not test the primary prediction of the lexical legacy hypothesis, that diversity should be beneficial when a semantic task requires flexible usage of a word, such as understanding or using a word in a new context.

One issue with natural language processing research is that it is difficult to control for all the potential variables that may affect lexical and semantic processing, such as word frequency, document count (one metric of contextual diversity), ambiguity, and imageability (McDonald & Shillcock, 2001). Word-learning studies can address these concerns. Johns et al. (2016) presented participants with pseudowords in passages focused on five topics (e.g., symptoms, stars, sociology, images, and stresses) or just one topic, through which they could infer their meaning (e.g., constellation). In line with natural language processing studies, words seen in high relative to low diversity contexts had higher accuracy and faster reaction times on a post-test old-new decision task, in which participants had to judge whether an item was one they had learned or not, but were less accurate in judging whether a learned word’s meaning was similar to a close synonym.

Bolger et al. (2008) exposed participants to rare English words either in four sentences that each described a new context or in the exact same sentence four times. In contrast to Johns et al. (2016), they found that words experienced in diverse relative to repeated sentence contexts subsequently showed more accurate meaning generation and quicker sentence completion. This may be because these tasks came closer to assessing generalisation of meaning knowledge rather than precise knowledge of a specific meaning. This idea is supported by the fact that Bolger et al. found that the high diversity advantage in these tasks was only present when definitions were not provided alongside sentences during training, i.e., when participants had to extract a general meaning of the word from the sentence contexts they had read. Supporting findings were reported by Pagán and Nation (2019). Using eye-tracking, they showed that words learned in varying rather than repeated sentence contexts (which provided cues to word meaning) were subsequently read faster in neutral sentences that were not informative as to word meaning. They argued that diversity during learning leads to better identification and integration when words are experienced in new contexts. However, one issue with both this and Bolger et al.’s study is that high diversity was compared to no diversity, i.e., repeated sentences, rather than low diversity. This may have an unintended consequence of reducing participants’ attention in the low diversity condition since repeated sentences are likely to be less interesting to read.

Joseph and Nation (2018) included a true low diversity condition in their experiment. Children read six unfamiliar English verbs (e.g., amalgamated) embedded in a series of short sentences that were either low (1 topic) or high (10 topics, e.g., law, medicine, money, work) in contextual diversity. They also explicitly probed generalisation of word meaning knowledge using a cloze task in which participants completed a sentence using one of the six target words. For half the trials, the sentence was derived from a context experienced during training, thus requiring little generalisation, whereas for the other half of the trials the sentences were derived from a new unseen context, thus requiring participants to generalise their knowledge of the word. However, no effect of diversity was observed in this task, nor in a spelling test that assessed word form learning. Since existing vocabulary size helps to scaffold the integration of new words (James et al., 2017) children may be worse than adults at inferring the meaning of words through context (Cain et al., 2004) leading to these null results.

Norman et al. (2022) therefore used Joseph and Nation’s (2018) stimuli and cloze task with adults. However, they replaced the English words with pseudowords, to ensure that participants had no pre-existing knowledge of their meanings or forms, and added two extra items (designed for but not used in the original study) to increase power. In the cloze task, they found that accuracy was higher for words learned in low relative to high diversity contexts for cloze sentences drawn from a context experienced during training. However, for cloze sentences drawn from a new unseen context, accuracy was higher for words learned in high relative to low diversity contexts. This is in line with the ideas expressed by the lexical legacy and lexical quality hypotheses, that contextual diversity should help readers to generalise and use a word in new contexts. However, in an old-new decision task that assessed word form knowledge, Norman et al. obtained no effect of contextual diversity. This is consistent with some other studies with adults that have also found no effect of contextual diversity on word form learning (Bolger et al., 2008; Hulme et al., 2023).

Mak et al. (2021) conducted two experiments that suggested that the benefit of contextual diversity may depend on new words being “anchored,” by first experiencing them in a restricted context to create a stable semantic representation, before being encountered in more diverse contexts that create a richer representation. In Experiment 1, they exposed adults to 10 pseudowords that were associated with the meanings of low-frequency words (e.g., mendacious). Five were learned in six passages spanning different topics (high diversity, e.g., Brexit, David Bowie, and Donald Trump), and five were learned in six passages focused on the same topic (low diversity). Words learnt in the low diversity condition showed better word form learning, indexed by higher old-new decision accuracy, as well as word meaning learning, indexed by more accurate semantic relatedness judgements. Mak et al.’s Experiment 2 then incorporated an “anchoring” opportunity, whereby new words were first presented in five sentences focused on the same topic (anchoring phase) and were then encountered three more times (post-anchoring phase) in either the same topic (low diversity) or one new topic (high diversity). Unlike in Experiment 1, word form learning was better for high relative to low diversity items, as seen by faster old-new decision responses. Moreover, the low diversity advantage for meaning knowledge was no longer present.

Mak et al. (2021) conducted semantic network simulations to understand the representational differences between high- and low-diversity words. Simulations 1 and 2 mirrored Experiments 1 (no-anchoring) and 2 (anchoring), respectively, with “training” being instantiated by creating a corpus of words from all passages associated with a particular trained item. Across both simulations, low-diversity words had a denser local neighbourhood of words with which they were connected, whereas high-diversity words received activation from more unique word nodes. In Simulation 1, the benefit of the former over-rode any benefit of the latter, resulting in more activation for low diversity words, interpreted as greater “efficiency with which the word was retrieved from the lexicon.” (p9). However, in Simulation 2, the corpus for high-diversity words also contained a high number of words from the same context (anchoring), in addition to a number of words from a different context (post-anchoring). This resulted in high-diversity words receiving more overall activation than low-diversity words.

Mak et al. (2021) argued that high-diversity words require a sufficiently dense local neighbourhood to benefit from activation from additional unique word nodes. They therefore proposed that restricting contextual diversity in early word learning, or “anchoring,” can help secure new items in memory and make them more salient and accessible. However, high diversity in later word learning increases the breadth of word meaning knowledge, both decontextualising word representations (supporting recognition across contexts or in isolation) and supporting the ability to understand and use words in different contexts. This view was echoed in a recent review by Raviv et al. (2022), which collated research across different fields and argued that variability differentially affects initial learning and generalisation. However, a key limitation of Mak et al.’s study was that across the anchoring and post-anchoring phases in Experiment 2, high diversity items were only seen in two topics, which is far fewer than the high diversity condition in Experiment 1 and in previous studies.

### The present experiment

The present study builds on Mak et al. (2021) and Norman et al. (2022) to further explore the potential benefits of anchoring for word form and meaning learning. In addition, we hope to replicate the key result from Norman et al., that high relative to low diversity contexts during learning facilitates generalisation of meaning knowledge in a cloze task. Adults learned eight pseudowords, each of which was first presented in five sentences, all drawn from one topic (anchoring phase). High diversity items were then presented in five further sentences, each from a different topic, whereas low diversity items were presented in five further sentences from the same topic as in the anchoring phase (post-anchoring phase). As in Norman et al., learning was assessed using an old-new decision task and a cloze task. Results were analysed for the current study and were also compared to those of Norman et al., as described in the hypotheses. The experiment was preregistered through AsPredicted (https://aspredicted.org/4ZD_N4R).

### Hypotheses

**1a. Contextual diversity will benefit word form learning.** We predicted that words experienced in high contextual diversity would show higher accuracy and faster response times (RTs) on the old-new decision task compared to words experienced in low contextual diversity. Though Norman et al. (2022) found no effect of diversity on old-new decision, we expected to due to the introduction of the anchoring phase (Mak et al., 2021).
**1b. Contextual diversity will benefit generalisation of word meaning knowledge.** In the cloze task, we predicted an interaction between cloze type and contextual diversity, as in Norman et al. (2022). Specifically, we predicted that for sentences drawn from a context not experienced during training (new), accuracy would be higher for high relative to low diversity items, while for sentences drawn from a context that was experienced during training (old), performance would not differ between high and low diversity items. Although Norman et al. obtained a benefit for low relative to high diversity items for old sentence types, we did not expect this in the current study since the high diversity items were experienced five times in the old context during the anchoring phase (as opposed to only once in Norman et al.).
**2a. An initial anchoring phase will enhance the contextual diversity benefit on word form learning.** By comparing our results with those of Norman et al. (2022), which offered no anchoring opportunity, we were able to further ascertain how anchoring modulates contextual diversity effects. We predicted an interaction between experiment (anchoring/non-anchoring) and diversity (high/low) in the old-new decision task such that in the current experiment (with anchoring) there would be a benefit for high relative to low diversity items in accuracy and RTs, whereas in Norman et al. (non-anchoring) there was no effect of diversity on performance.
**2b. An initial anchoring phase will enhance contextual diversity benefits on word meaning learning.** For the cloze task, Norman et al. (2022) found a crossover interaction between contextual diversity and cloze type. Although we also expected to find this interaction, we predicted that the nature of the interaction would differ between experiments. We therefore predicted a three-way interaction between experiment (anchoring/non-anchoring), cloze type (old/new context), and diversity (high/low), such that anchoring would enhance the high diversity advantage found for new cloze sentence types and reduce the low diversity advantage seen for old sentence types.

## Method

### Ethics

Ethical approval was obtained from the University College London Language and Cognition Departmental Ethics Committee (Project ID: LCD-2020-02), and participants gave their informed consent before taking part.

### Participants

A total of 288 participants were recruited, to match Norman et al.’s (2022) sample (*N* = 260). The sample size approaches, although does not reach, the 1600 observations per condition (260 participants × 4 stimuli = 1040) recommended to achieve 80% power in mixed-effects analyses (Brysbaert & Stevens, 2018) and is a far larger sample than in any previous word learning study on contextual diversity. All participants were recruited through Prolific (www.prolific.co). They were recruited in two separate batches of 101 and 187 participants, in July 2021 and May 2022 respectively, and paid in accordance with institutional requirements (£7.50/hour in 2021, £9/hour in 2022). Ten participants were excluded from data analysis as they either reported writing notes during the experiment (*N* = 3), or scored at or below chance on the comprehension questions during the learning phase, that is, 4 or fewer questions out of 8 correct (*N* = 7). All participants were native English speakers with no reported visual, language, or hearing impairments. There were no requirements for participants to be monolingual English speakers (see Appendix A in the online Supplementary Material for the full breakdown of demographic details). All analyses were conducted based on the remaining 278 participants (*M_age_* = 28.11 years, *SD_age_* = 6.35 years; *N_female_* = 173, *N_male_* = 100, *N_non-binary_* = 5).

### Design

Contextual diversity (low vs. high) was manipulated within-subjects and within-items. The number of contexts experienced for each pseudoword was either one (low diversity) or six (high diversity). Participants read half of the pseudowords in the low diversity condition, and half in the high diversity condition. The contextual diversity of pseudowords as well as their underlying meaning was counterbalanced across participants.

Anchoring was manipulated between-subjects and within-items, such that participants in the current experiment experienced an anchoring phase whereas those in Norman et al. (2022), to which our data will be compared, did not. In the current experiment, the learning phase was separated into an anchoring and post-anchoring phase. Table 1 explains the anchoring and diversity manipulations across the two experiments. It can be seen that participants experienced fewer contexts in the high diversity condition in the current experiment as compared to Norman et al. This is because it was not possible to simultaneously equate number of contexts and frequency of exposure across the two experiments and we deemed it more important to balance frequency given its known effect on word learning and processing. Overall, participants in both experiments read 80 sentences and experienced each pseudoword 10 times across the learning phase.

**Table 1.** Manipulation of contextual diversity and anchoring across the learning phase.

	High diversity		Low diversity	
	Sentences 1–5	Sentences 6–10	Sentences 1–5	Sentences 6–10
Anchoring experiment (Current study)	Context 1 (anchoring)	Contexts 2–6 (post-anchoring)	Context 1 (anchoring)	Context 1 (post-anchoring)
No-anchoring experiment (Norman et al., 2022)	Contexts 1–10		Context 1	

Word form learning was measured as the accuracy and correct RTs for trained items on the old-new decision task. Word meaning learning was measured as accuracy on the cloze task. Cloze sentence type was manipulated within-subjects: participants were tested with a new sentence context and an old sentence context for each item.

### Materials

#### Target words and associated pseudowords

Stimuli were taken from Norman et al. (2022), who adapted items used in Joseph and Nation (2018). In Joseph and Nation’s study, children learned the meanings of six low-frequency past tense verbs (e.g., amalgamated). Norman et al. included two additional words to increase the power of the study, resulting in eight target words. These words (and their associated training sentences) were created by Joseph and Nation but not used in their final stimulus set. As stated by Joseph and Nation (p193) “Verbs allowed us to move beyond the word–object referent mapping tasks. . . to consider words that have more complex and nuanced meanings.” This means that the items are akin to the types of new words adults would encounter when reading natural texts. To ensure adult participants had no pre-existing knowledge of the items, the words were replaced with pseudowords (e.g., lindered). Table 2 shows the pseudowords, which were adapted from Hulme et al. (2022), and Norman et al. (2022) provides a fuller description of how these items were created. The two columns in Table 2 show how pseudoword-to-meaning assignment was counterbalanced across participants.

**Table 2.** Full list of target words and pseudoword replacements in the counterbalanced item lists.

Word meanings	Pseudoword assignment 1	Pseudoword assignment 2
Accumulated	invilled	rudgerbed
Amalgamated	lindered	uzided
Intervened	rudgerbed	invilled
Exacerbated	uzided	lindered
Confabulated	sottled	danested
Languished	noffled	perphised
Divulged	perphised	noffled
Thwarted	danested	sottled

#### Sentence contexts for the learning phase

The target words were embedded in high- and low-diversity sentence contexts. These sentences were all of similar length, and target words were never the first or last word of the sentence. For the current experiment, 120 sentences in total were selected, 80 low diversity and 40 high diversity. Fewer high-diversity sentences were used in our study than in Norman et al. (2022) since only low-diversity sentences were used in the anchoring phase. For each participant, four pseudowords were experienced in the low diversity condition, in which all sentences describe the same context (e.g., all about animals), while the other four were experienced in the high diversity condition, in which each sentence described a different context (e.g. animals, law and schools). Appendix B in the online Supplementary Material provides the experimental sentences used in the learning phase. Pseudoword-to-diversity condition assignment was counterbalanced across participants.

#### Stimuli for the testing phase

For the old-new decision task, half of the trials consisted of foils which were one-letter different to the target pseudowords. Like the trained items, these were taken from Norman et al. (2022), who selected them from a word learning study by Hulme et al. (2022). Hulme et al. verified that the foils and trained items were of equivalent word-likeness. Table 3 shows the trained pseudowords and matched foils and a fuller description of these stimuli is provided in Norman et al. Sentences for the cloze task were taken from Joseph and Nation (2018). For each item there was one sentence drawn from the same context experienced in the anchoring phase, though not seen previously (old sentence), and one sentence that described a new context not encountered during learning (new sentence). This allowed us to examine the extent to which participants could generalise their learning to a new context (see Appendix C in the online Supplementary Material for the sentences used in the cloze task).

**Table 3.** Target and foil stimuli for the old-new decision task.

Trained pseudoword	Foil
Invilled	Invilted
Lindered	Lundered
Sottled	Sittled
Noffled	Naffled
Perphised	Perprised
Rudgerbed	Rudgerded
Uzided	Uzibed
Danested	Danepted

### Procedure

#### Learning phase

**Figure 1.**
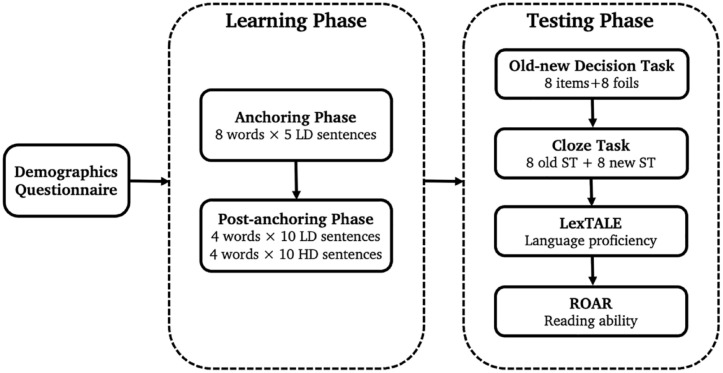
Diagram of the experimental procedure.

Figure 1 illustrates the full procedure. The experiment was conducted using Gorilla Experiment Builder (www.gorilla.sc; Anwyl-Irvine et al., 2020). The experimental tasks have been made available on Gorilla Open Materials and can be accessed at the following link: https://app.gorilla.sc/openmaterials/612824. Participants were randomly assigned to one of the four counterbalanced versions of the experiment such that there were equal numbers of participants assigned to each version (not accounting for subsequent exclusions). They completed the main reading task, in which sentences were presented on screen one at a time (see Appendix D in the online Supplementary Material for the full list of instructions used in the experiment). No time limit was imposed but reading time was recorded. During the anchoring phase, participants read five blocks of eight sentences (one sentence for each pseudoword), all drawn from the low diversity condition. The order of trials within blocks and the block order were randomised. In the post-anchoring phase, participants read five more blocks of eight sentences (one sentence for each pseudoword); for half the items, these sentences were drawn from the high diversity condition and for half the items, they continued to be drawn from the low diversity condition (though were new sentences). Comprehension questions after occasional items across both anchoring and post-anchoring phases ensured that attention was maintained. There were eight comprehension questions in total (one for each pseudoword) and they required a true or false response. These questions were related to the content of the sentence but not to the meaning of the to-be-learned pseudoword. Participants were not informed of the transition from the anchoring to post-anchoring phase, but an optional break was offered to participants once in each phase.

#### Testing phase

Following the learning phase, participants completed two tasks to measure their pseudoword knowledge. In the old-new decision task, participants were told they would see a series of words, some of which they had encountered in the previous task, and others that would be spelled incorrectly. They should judge as quickly and as accurately as possible whether the word on the screen is spelled correctly or incorrectly, by pressing either the “f” or “j” key on their keyboard, respectively. There were 16 trials presented in a randomised order, 8 for the correct pseudowords and 8 foils.

In the cloze task, participants saw a series of sentences, one at a time. Each of the sentences had a missing word (e.g., “The police ________ evidence against the suspect at a very rapid pace.”). They were asked to select the correct response from one of the eight trained pseudowords, which were displayed on screen at all times. There were two practice questions with real English words to familiarise participants with the task, for which feedback was provided. No feedback was given in the main task. For each item there was one sentence drawn from an old context and one sentence drawn from a new context, and thus 16 trials in total, presented in a randomised order. Participants were told that there was more than one sentence that goes with each word.

After completing both post-tests, participants did adapted versions of the Lexical Test for Advanced Learners of English (LexTALE; Lemhöfer & Broersma, 2012) and the Rapid Online Assessment of Reading Ability (ROAR; Yeatman et al., 2021). The LexTALE is an un-speeded lexical decision task, consisting of 60 trials (40 real words and 20 pseudowords) presented in a randomised order. Each trial started with a 200 ms fixation cross, followed by a letter string that was displayed until participants pressed “f” to indicate the string is a word, or “j” to indicate that it is not. A second fixation cross was then displayed for 200 ms before the next trial began. Scores were calculated as: (proportion of words correct + proportion of pseudowords correct) / 2. LexTALE score is reported to be a valid measure of vocabulary knowledge and language proficiency for non-native English speakers. The ROAR is a speeded lexical decision task consisting of 42 words and 42 pseudowords presented in a randomised order. There were three different lists of items with each participant just completing one list as selected by the experimental software. Each trial began with a 400 ms fixation cross, after which a letter string was displayed for 350 ms, followed by a blank screen that remained until participants pressed “f” to indicate the string was a word, or “j” to indicate that it was not. The blank screen then continued for 100 ms before the next trial started. Total accuracy on the ROAR is a reliable measure of reading ability in developmental samples (Yeatman et al., 2021). We acknowledge that both these measures may show a limited distribution with the native English-speaking adults in the current experiment. We therefore also analysed response time on the ROAR. While this has not been confirmed to be a reliable measure of individual differences, Yeatman et al. did find that response time correlated with reading ability assessed using standardised measures in higher performing children and adults.

### Data analyses

Analyses were conducted using *R* (version 4.1.2; R Core Team, 2022) using the lme4 package (Bates et al., 2015). Linear mixed-effects models were used to analyse the RTs in the old-new decision task, while accuracy in the old-new decision and the cloze task were analysed using generalised linear mixed-effects models. Deviation coding was used to define the contrasts for the fixed effects of Diversity (high: 0.5 vs. low: −0.5), Cloze Type (new: 0.5 vs. old: −0.5), and Experiment (anchoring experiment: 0.5 vs. non-anchoring experiment: −0.5), with the interactions coded by multiplying the contrasts for the relevant factors. To determine the appropriate random effects structure for each model, we first constructed the maximal model (Barr et al., 2013), which contained by-participant and by-item random intercepts, along with by-participant and by-item random slopes for all factors of interest. In our analyses, however, the maximal structure either failed to converge or resulted in a singular fit. Therefore, all models were progressively simplified following procedures suggested by Barr et al. (2013) until reaching convergence (without overfitting).

We first removed the correlations between random intercepts and random slopes. Unless the model converged (without overfitting), the random by-item and by-participant intercepts were then dropped from the model (with all random slopes kept in). If this model still had convergence issues, we adopted a data-driven forward model selection strategy starting with the simplest model with by-item and by-participant random intercepts only and adding in each of the random slopes one at a time. Any models that converged from this selection process were then tested for inclusion independently against the simple random intercepts only model using likelihood ratio tests (Barr et al., 2013; Matuschek et al., 2017). If none of these models provided a better fit to the data relative to the random intercepts only model (*p* > .2), the simplest model was used as the final model. Otherwise, the model containing the single random slope with the lowest *p-*value was selected and then compared against the other converged models containing this slope and a second slope. This procedure of testing goodness of fit was repeated, taking the model with the lowest *p*-value in each case until all the slopes were tested for inclusion and there was no significant improvement. For the generalised linear mixed-effects models we used the BOBYQA (Bound Optimisation BY Quadratic Approximation) optimiser to facilitate model convergence (Bates et al., 2021).

To assess the significance of the fixed effects and interactions, we used likelihood ratio tests to compare the final model (including all fixed effects) to models with the relevant fixed effect or interaction removed. For any significant interaction, follow-up simple effects analyses were run, in which the data were subsetted according to one of the fixed effects and we followed the same procedures as described for the main analysis to achieve model convergence and assess the significance of the remaining fixed effects. The final model that was selected was always the most complex model that converged for all subsets. The significance level of these simple effects analyses was adjusted using the Bonferroni correction method.

## Results

### Anchoring experiment

#### Learning task

The mean percentage of comprehension questions answered correctly for the 278 included participants was 87.10% (*SD* = 11.26%). This did not differ for high (*M* = 87.23%, *SD* = 16.14%) diversity items as compared to low (*M* = 86.96%, *SD* = 17.09%) diversity items.

#### Old-new decision task

For the analysis of reaction times (RTs), only correct responses were analysed. These were visually inspected using a histogram, and extreme outlier responses (exceeding 9000 ms) were excluded from the analysis (*n* = 6). To check the assumptions of normality and homoscedasticity, a histogram of the residuals and a scatterplot of the residuals vs. fitted values were created. Inspection of these figures revealed a violation of assumptions, so log and inverse (1000 ms / raw RT) transformations were applied. As the inverse-transformed RTs more closely met the assumptions of homoscedasticity, these were used in the data analysis.

##### Trained versus foil items

To determine the validity of our learning paradigm, we first compared performance on the trained stimuli with untrained foils, with the expectation of faster and more accurate responses to the trained items, as is typical for words relative to pseudowords in lexical decision tasks. In the models that converged for both RT and accuracy, stimulus type (trained item vs. foil item) was the fixed factor, and the random effects structure contained random intercepts for participants and items and a by-participant random slope for stimulus type. As expected, accuracy was significantly higher, *χ*^2^(1) = 8.37, *p* = .004, for trained items (*M* = 91.5%, *SD* = 27.9%) than foils (*M* = 79.3%, *SD* = 40.5%), and responses were significantly faster, *χ*^2^(1) = 11.34, *p* < .001, for trained items (*M* = 1163.04 ms, *SD* = 878.69 ms) than for foils (*M* = 1321.11 ms, *SD* = 1049.79 ms).

##### High versus low diversity

**Figure 2.**
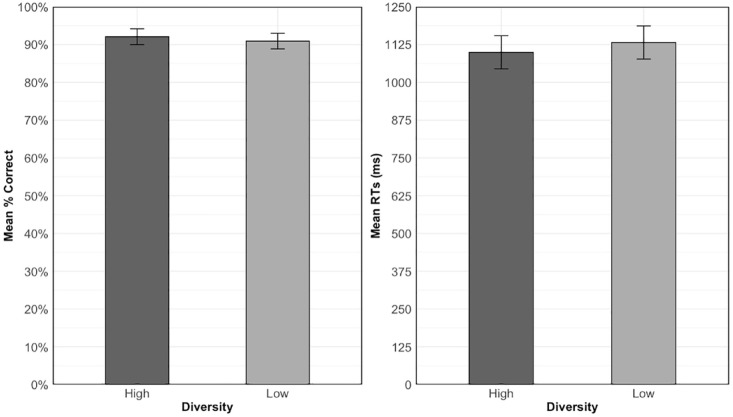
Mean accuracy (left) and RTs for correct responses (right) for trained items across diversity conditions in the old-new decision task. Error bars show the standard error of the means, adjusted for the within-participant design (Cousineau, 2005).

Accuracy and RTs for trained items are shown in Figure 2. These were analysed to test Hypothesis 1a: experiencing words in high versus low diversity contexts will lead to better performance on the old-new decision task. The final model for the accuracy analysis was the random intercepts only model while the RT model contained an additional by-participants random slope for diversity. Table 4 shows the results of these analyses. The data for accuracy showed no significant effect of diversity, *χ*^2^(1) = 1.12, *p* = .290. Participants exhibited a high level of accuracy in both the high (*M* = 92.1%, *SD* = 27%) and low (*M* = 90.9%, *SD* = 28.7%) diversity conditions. Despite numerically faster RTs for items learned in high (*M* = 1099.54 ms, *SD* = 602.12 ms) relative to low diversity contexts (*M* = 1131.79 ms, *SD* = 781.23 ms), this effect was also non-significant, χ^2^(1) = 0.003, *p* = .956.

**Table 4.** Results of the generalised linear mixed-effects models for anchoring experiment data.

Task (measure)	Fixed effects	Est/Beta	*SE*	t/z	χ^2^	*p*
Old-new decision (Accuracy)	(Intercept)	2.36	0.19	12.15	—	—
	Item Type (stimuli vs. foil)	1.18	0.38	3.14	8.37	.**004**
Old-new decision (RTs)	(Intercept)	< .01	< .01	53.45	—	—
	Item Type (stimuli vs. foil)	< .01	< .01	4.06	11.34	**<** **.001**
Old-new decision (Accuracy)	(Intercept)	2.94	0.15	19.22	—	—
	Diversity	0.17	0.16	1.06	1.12	.290
Old-new decision (RTs)	(Intercept)	< .01	< .01	46.87	—	—
	Diversity	< .01	< .01	0.06	0.00	.956
Cloze (Accuracy)	(Intercept)	0.57	0.25	2.31	—	—
	Diversity	−0.13	0.07	−1.71	2.84	.092
	Cloze Type	−0.62	0.23	−2.70	5.18	**.023**
	Diversity × Cloze Type	0.65	0.15	4.47	19.56	**<** **.001**

RTs: response times. *Note.* The *p-*values of significant fixed effects are presented in bold.

#### Cloze task

**Figure 3.**
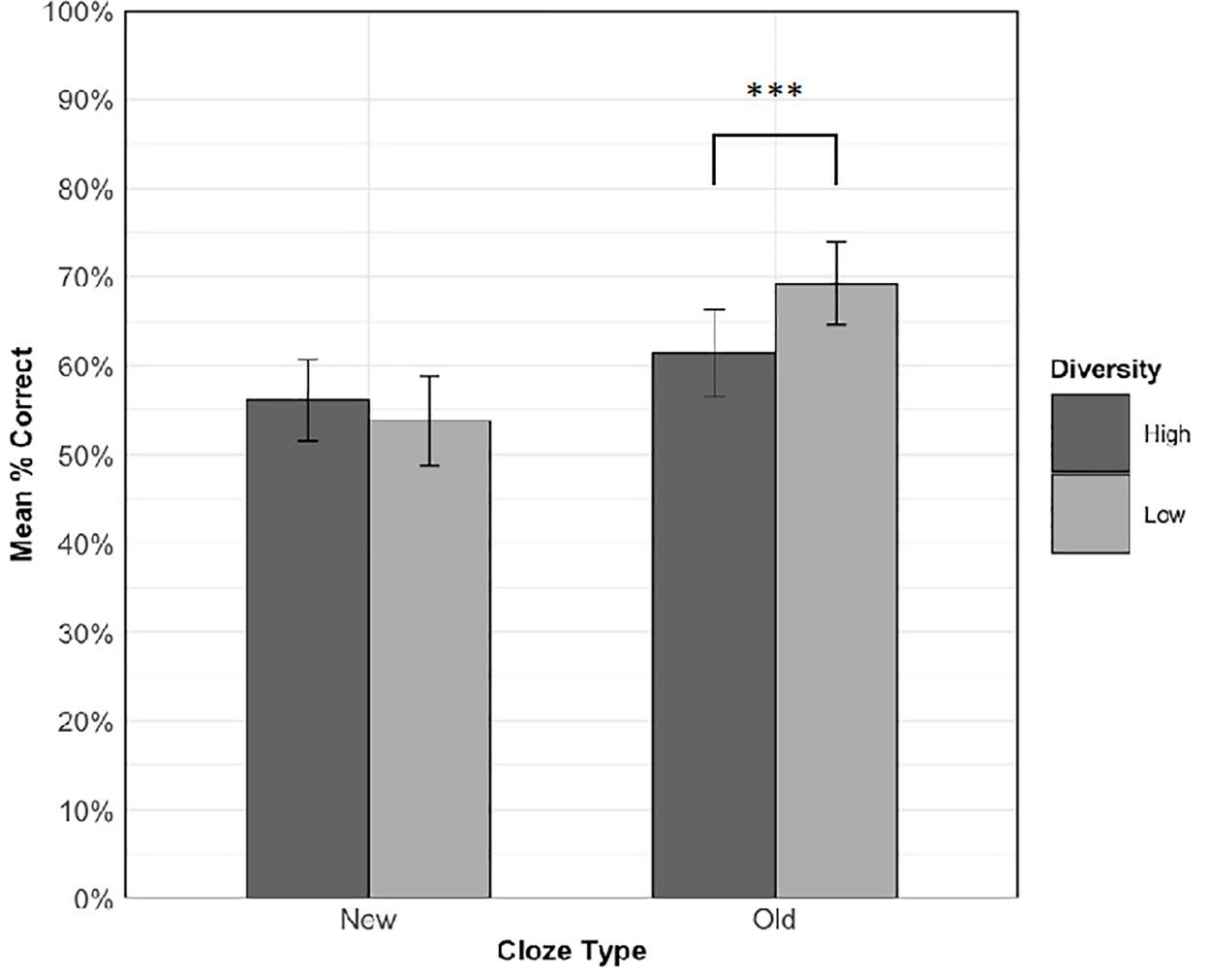
Mean accuracy on the cloze task across diversity conditions for each cloze type. Error bars show standard error of the means, adjusted for the within-participant design (Cousineau, 2005). ***p < .001, α =.025.

Figure 3 shows the mean accuracy for each condition in the cloze task. Overall accuracy was above chance (*M* = 60.1%, *SD* = 25.3%, chance = 12.5% or 1/8), which indicates that participants were able to gain some semantic knowledge of the pseudowords. Hypothesis 1b predicted an interaction between cloze type and diversity. Specifically, we expected that on new sentence types, accuracy would be higher for items experienced in high relative to low diversity contexts, but that there would be no diversity effect for old sentence types. To test this hypothesis, diversity (high vs. low), cloze type (old vs. new), and the interaction were entered into the model as fixed effects of experimental interest. The random effects structure contained random intercepts for participants and items, and a by-item random slope for cloze type. Table 4 shows the results of this analysis.

There was a significant main effect of cloze type, *χ*^2^(1) = 5.18, *p* = .023, with higher accuracy for old sentences than new sentences. No significant main effect of diversity was observed, *χ*^2^(1) = 2.84, *p* = .092. As predicted, there was a significant interaction between cloze type and diversity, *χ*^2^(1) = 19.56, *p* < .001, so follow-up simple effects analyses were conducted to examine diversity effects in old and new sentences separately (α = .025, Bonferroni-corrected). The final models for these subset analyses contained random intercepts for participants and items and a by-item random slope for cloze type, along with the fixed effect of diversity.

Contrary to our hypothesis, for cloze sentences drawn from old, familiar contexts, accuracy was significantly higher, *χ*^2^(1) = 15.82, *p* < .001, for words learned in low (*M* = 69.2%, *SD* = 46.2%) relative to high (*M* = 61.4%, *SD* = 48.7%) diversity contexts. However, for cloze sentences drawn from new unfamiliar contexts, accuracy was numerically, but non-significantly, *χ*^2^(1) = 3.58, *p* = .058, higher for words learned in high (*M* = 56.1%, *SD* = 49.6%) relative to low (*M* = 53.8%, *SD* = 49.9%) diversity contexts.

### Anchoring experiment vs. non-anchoring experiment

#### Old-new decision task

**Figure 4.**
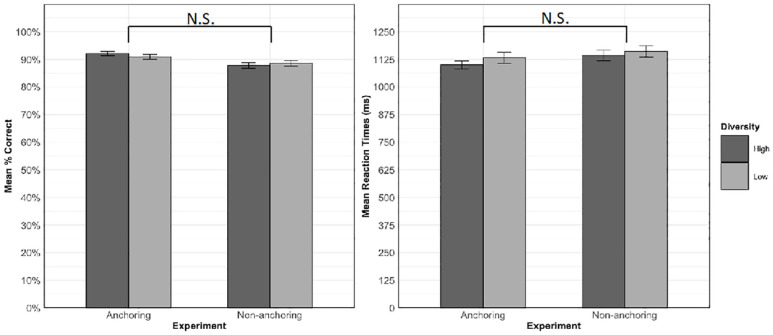
Mean accuracy (left) and RTs for correct responses (right) to trained items on the old-new decision task in the high and low diversity conditions in the two experiments. Error bars represent ± 1 standard error from the mean. N.S. = not significant.

We next compared the data from the current experiment, in which training included an anchoring phase before diversity was introduced, with data from Norman et al. (2022), which did not include an anchoring phase and introduced diversity from the beginning of training. Hypothesis 2a predicted that high relative to low diversity items would experience a greater benefit in old-new decision accuracy and RTs in the current versus the previous experiment. Mean accuracy and RTs for trained items in each condition in each experiment are shown in Figure 4. The models for the old-new decision data analysis included diversity, experiment (anchoring vs. non-anchoring), and the diversity by experiment interaction as fixed effects. The random-intercepts only model was selected for the analysis of accuracy and RT. Table 5 shows the results of these analyses.

**Table 5.** Results of the (generalised) linear mixed-effects models for examining effects of diversity and experiment in the old-new decision data.

Measure	Fixed effects	Est/Beta	SE	t/z	χ^2^	*p*
Accuracy	(Intercept)	3.02	0.13	22.38	—	—
	Diversity	0.04	0.12	0.32	0.10	.751
	Experiment	0.35	0.19	1.79	3.17	.075
	Diversity × Experiment	0.30	0.24	1.27	1.62	.204
RTs	(Intercept)	< .01	< .01	43.53	—	—
	Diversity	< .01	< .01	0.63	0.39	.532
	Experiment	< .01	< .01	1.28	1.63	.202
	Diversity × Experiment	< .01	< .01	-0.45	0.20	. 651

*Note.* The *p-*values of significant fixed effects are presented in bold.

Contrary to Hypothesis 2a, there was no significant interaction between experiment and diversity in the accuracy, χ^2^(1) = 1.62, *p* = .204, or RT, χ^2^(1) = 0.20, *p* = .651, analysis. There was no main effect of diversity on either accuracy, χ^2^(1) = 0.13, *p* = .714, or RTs, χ^2^(1) = 0.25, *p* = .619. Although participants in the anchoring experiment responded more accurately and faster compared to the participants in the non-anchoring experiment, the main effect of experiment was also non-significant for both accuracy, χ^2^(1) = 3.17, *p* = .075, and RTs, χ^2^(1) = 1.63, *p* = .202.

#### Cloze task

**Figure 5.**
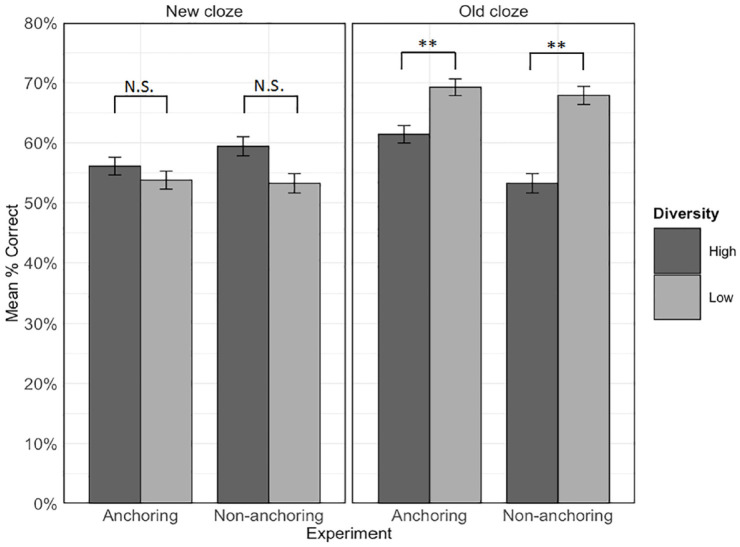
Mean accuracy in each diversity condition within new and old cloze sentences in each experiment. Error bars represent ± 1 standard error from the mean. **p < .01, corrected. N.S. = not significant; α = .0125.

For the cloze task, Hypothesis 2b predicted a three-way interaction between experiment, diversity, and cloze type. Specifically, we expected that there would be a greater benefit for the high relative to the low diversity condition for new cloze sentences in the anchoring versus the non-anchoring experiment, and, conversely, a greater benefit for the low relative to the high diversity condition for old cloze sentences in the non-anchoring versus the anchoring experiment. Figure 5 shows mean accuracy in each condition in each experiment. The generalised linear mixed-effects model included the fixed effects of diversity, experiment, cloze type, and their two- and three-way interactions. The random effects structure contained random intercepts by participants and by-items, a by-participants random slope for diversity, and by-items random slopes for cloze type and experiment. Table 6 shows the results of this analysis. There were main effects of diversity and cloze type, as well as experiment x cloze type and diversity x cloze type interactions. Most importantly, as predicted, there was also a three-way interaction between diversity, cloze type, and experiment, χ^2^(1) = 6.92, *p* = .009, that is further explored in the next section.

**Table 6.** Results of the generalised linear mixed-effects models for examining effects of diversity, cloze type, and experiment in the cloze task.

Fixed effects	Est/Beta	*SE*	z	χ^2^	*p*
(Intercept)	0.60	0.22	2.71	—	—
Diversity	−0.17	0.07	−2.65	6.70	**.010**
Cloze Type	−0.43	0.17	−2.51	4.67	**.031**
Experiment	0.19	0.16	1.20	1.40	.236
Diversity × Cloze Type	0.93	0.11	8.61	72.62	< **.001**
Diversity × Experiment	0.12	0.13	0.96	0.89	.345
Cloze Type × Experiment	−0.33	0.11	−3.10	9.28	**.002**
Diversity × Cloze × Experiment	−0.57	0.21	−2.68	6.92	**.009**

Note. The p-values of significant fixed effects are presented in bold.”

##### Interaction between cloze type, diversity, and experiment

To understand the nature of the three-way interaction and test Hypothesis 2b—that the effect of diversity would differ between experiments for the two sentence types, we first examined the diversity by experiment interaction within each cloze type (Figure 5). The final model for each cloze type contained by-item and by-participant random intercepts, the by-item random slope for diversity, and fixed effects of diversity, experiment, and the diversity by experiment interaction. There was a significant interaction between diversity and experiment within old cloze sentences, χ^2^(1) = 7.06, *p* = .008; α = .025, Bonferroni-corrected, but not within the new cloze sentences, χ^2^(1) = 1.65, *p* = .199; α = .025.

To further understand these effects, the simple effects of diversity were then examined for each cloze type in each experiment separately. The full model for each analysis contained the by-item and by-participant random slopes and the fixed effect of diversity. As shown in Figure 5, when items were tested in old cloze types, accuracy was significantly higher in the low than the high diversity condition in both the non-anchoring, χ^2^(1) = 10.39, *p* = .001; α = .0125, Bonferroni-corrected; *M_high-diversity_* = 53.2%, *SD_high-diversity_* = 50%; *M_low-diversity_* = 67.9%, *SD_low-diversity_* = 46.7%, and anchoring, χ^2^ (1) = 7.06, *p* = .008; *M_high-diversity_* = 61.4%, *SD_high-diversity_* = 48.7%; *M_low-diversity_* = 69.2%, *SD_low-diversity_* = 46.2%, experiments. However, reflecting the significant interaction between diversity and experiment, the difference was smaller in the anchoring experiment, in line with our prediction that anchoring would reduce the benefit of low relative to high diversity for old sentence types. For new sentence types, there was no significant effect of diversity in either the non-anchoring, χ^2^(1) = 1.97, *p* = .161; *M_high-diversity_* = 59.4%, *SD_high-diversity_* = 49.1%; *M_low-diversity_* = 53.2%, *SD_low-diversity_* = 50.0%, or the current anchoring experiment, χ^2^ (1) = 0.42, *p* = .518, *M_high-diversity_* = 56.1%, *SD_high-diversity_* = 49.5%; *M_low-diversity_* = 53.8%, *SD_low-diversity_* = 50.1%. This finding is contrary to our prediction since anchoring did not boost the advantage of learning words in high diversity contexts for completing new cloze sentences.

**Figure 6.**
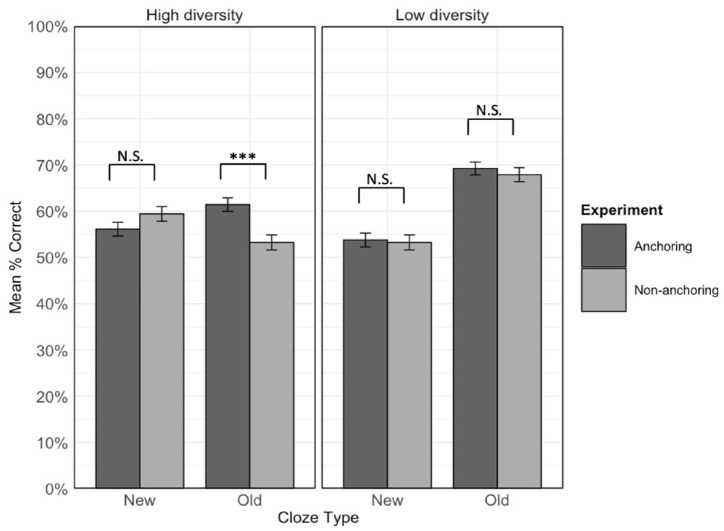
Mean accuracy in each experiment within each diversity condition for new and old sentence types. Error bars represent ± 1 standard error from the mean. ***p < .001, corrected, N.S. = not significant; α = .0125.

We further explored the three-way interaction by examining the cloze type by experiment interaction within high and low diversity conditions (Figure 6). The final model for each diversity condition contained by-item and by-participant random intercepts and the by-item random slope for cloze type, along with the fixed effects of cloze type, experiment, and the cloze type by experiment interaction. There was a significant interaction between cloze type and experiment in the high diversity condition, χ^2^(1) = 20.32, *p* < .001; α = .025, Bonferroni-corrected, but not in the low diversity condition, χ^2^(1) = 0, *p* = 1.

The simple effects of experiment were then examined within each cloze type for high and low-diversity items separately. The final model for each analysis contained the by-item and by-participant random intercepts and the fixed effect of cloze type. For items experienced in the high diversity condition, there was a significant effect of experiment for items tested in the old cloze type, χ^2^(1) = 11.17, *p* < .001; α = .0125, Bonferroni-corrected, but not the new cloze type, χ^2^(1) = 0.61, *p* = .436. Figure 6 shows that for old cloze sentences, accuracy for high-diversity items was greater in the current anchoring experiment (*M* = 61.4%, *SD* = 48.7%) compared to the previous non-anchoring experiment (*M* = 53.2%, *SD* = 49.9%). This is again in line with our prediction that anchoring would reduce the relative advantage of the low over the high diversity condition for old sentence types. For items experienced in the low diversity condition, the simple effect of experiment was not significant for items tested in either old, χ^2^(1) = 0.28, *p* = .599, or new, χ^2^(1) = 0.26, *p* = .611, cloze type sentences (Old cloze: *M_non-anchoring_* = 67.9%, *SD_non-anchoring_* = 46.7%; *M_anchoring_* = 69.2%, *SD _anchoring_* = 46.2%; New cloze: *M_non-anchoring_* = 53.2%, *SD_non-anchoring_* = 50.0%; *M_anchoring_* = 53.8%, *SD _anchoring_* = 50.1%).

#### Exploratory analyses

##### Contextual constraint of sentences

A separate study was conducted to try to determine to what extent word meaning, in addition to contextual diversity, varied between high- and low-diversity sentences (c.f. Bolger et al., 2008; Cevoli et al., 2021; Hoffman et al., 2022). Twenty monolingual native English speakers (*M* = 28.2 years, *SD* = 5.84; 14 females) completed a cloze task on all 120 experimental sentences. The target word of each sentence was replaced with a blank, and participants were asked to type in a word that best fit the sentence, while being reminded that the same word might be applicable to multiple sentences.

WordNet (Miller, 1995) was used to compute similarity scores between participants’ responses and the original target word (e.g., accumulated), ranging from 0 (unrelated) to 1 (identical to the target word). Responses that did not exist in the English vocabulary, or were not verbs, were excluded from the final analysis (unexpected responses, 2.8%). One participant was excluded due to low similarity score (< 50%) and too many unexpected responses (25% of responses).

A linear mixed-effects model, including diversity as a fixed effect and random intercepts for participants and items, indicated that responses were more similar to the target word for high (*M* = 0.67, *SD* = 0.20) relative to low (*M* = 0.64, *SD* = 0.21) diversity sentences. This suggests that the meanings of pseudowords were more predictable in the high relative to low diversity condition. To ensure this difference was not due to the difference in the variability of words produced in different conditions, the number of different responses for each word in high and low diversity sentences was calculated (variability rate: high diversity = 0.32; low diversity = 0.28). The analysis of variability in responses did not show evidence for a significant diversity effect (see Appendix E in the online Supplementary Material for the full report of the analyses of contextual constraint of sentences). The implications of these results are further considered in the “Discussion” section.

##### Individual differences in lexical experience

To investigate how individual differences in lexical experience may relate to word learning, exploratory analyses were carried out, only on data from the current experiment (these tasks were not included in Norman et al., 2022). Participants’ lexical proficiency and reading ability were measured using the LexTALE (LexTALE score: *M* = 89.9, *SD* = 8.5) and the ROAR (Accuracy/84: *M* = 75.7, *SD* = 7.05; RTs: *M* = 634.09 ms, *SD* = 185.82 ms). Due to the non-normal distribution of data (all Shapiro–Wilk tests had *p* < .05), Spearman’s non-parametric correlations were conducted. Included in this analysis were accuracy and RTs on the old-new decision task, accuracy on the cloze task, accuracy and RTs on the ROAR, and the LexTALE score. Table 7 shows the correlations. In addition to a significant correlation between the accuracy scores of the two language proficiency tests, the results also demonstrated that accuracy on the ROAR and the LexTALE score were significantly and positively correlated with accuracy on the old-new decision task and the cloze task. There was also a positive correlation between the RTs of the old-new decision task and the ROAR.

**Table 7.** Nonparametric correlation coefficients comparing the relationship between language proficiency measures and word learning performance.

Variable	1	2	3	4	5	6
1. Old-new decision accuracy	—	0.11	**0.61**	**0.28**	0.02	**0.37**
2. Old-new decision RTs	0.11	—	0.01	**0.12**	**0.43**	−0.02
3.Cloze accuracy	**0.61**	0.01	—	**0.2**	0.03	**0.39**
4.ROAR scores	**0.28**	**0.12**	**0.2**	—	**0.4**	**0.41**
5.ROAR RTs	0.02	**0.43**	0.03	**0.4**	—	−0.1
6. LexTALE scores	**0.37**	−0.02	**0.39**	**0.41**	−0.07	—

RTs: response times; ROAR: rapid online assessment of reading ability; LexTALE: lexical test for advanced learners of English.

*Note*. Coefficients printed in bold are significant at *p* < .05.

## Discussion

This study tested whether an anchoring phase, in which all new words are first experienced in one context, increases the benefit of subsequent contextual diversity for word form and meaning learning. In addition, we hoped to replicate the key result from Norman et al. (2022), that high relative to low diversity contexts during learning facilitates generalisation of meaning knowledge in a cloze task. We now address whether results were in line with our specific hypotheses.

### Hypothesis 1a. Contextual diversity will benefit word form learning

Our first prediction was that words learned in sentences with high relative to low contextual diversity would show more accurate and faster responses on the old-new decision task. Our results did not support this prediction since we found no significant effect of contextual diversity on accuracy or reaction time in this task. Thus, our results mirror those obtained by Norman et al. (2022), despite the inclusion of anchoring in our experiment.

### Hypothesis 1b. Contextual diversity will benefit generalisation of word meaning knowledge

For the cloze task, which assessed word meaning learning, we expected to replicate Norman et al. (2022) and obtain an interaction between cloze type and diversity. Specifically, for cloze sentences drawn from new contexts, we predicted higher accuracy for high than low diversity items, but for cloze sentences drawn from an old context, we predicted no diversity effect. Although we did obtain the predicted interaction and the overall pattern of results was the same as in Norman et al., the simple effects did not support these predictions since the high diversity benefit for new cloze sentences was non-significant, while for old cloze sentences, accuracy was significantly higher for items in the low than the high diversity condition.

### Hypothesis 2a. An initial anchoring phase will enhance the contextual diversity benefit on word form learning

To determine whether anchoring induces a benefit of contextual diversity on word form learning (Mak et al., 2021), we compared our old-new decision data with those from Norman et al. (2022), which used the same training and testing paradigm but did not include an anchoring phase at the beginning of training. We predicted an interaction between experiment and diversity, in that the benefit for high relative to low diversity items would be greater in the current anchoring experiment than the non-anchoring experiment. However, we did not observe this; as in Norman et al., there was no effect of diversity on old-new decision accuracy or RTs in the current experiment.

### Hypothesis 2b. An initial anchoring phase will enhance contextual diversity benefits on word meaning learning

We expected to find a three-way interaction in the cloze task between experiment, diversity, and cloze type. That is, the benefit of the high relative to the low diversity condition for new sentences would be greater in the current experiment that included an anchoring opportunity, but the benefit of the low relative to the high diversity condition for old sentences would be greater in the previous non-anchoring experiment. The predicted three-way interaction was significant. However, follow-up analyses showed that for new sentences, the advantage for high relative to low diversity items was not significant in the current anchoring experiment, whereas Norman et al. (2022) obtained a significant benefit of diversity for new sentences. This contradicts the first part of our prediction and indicates no benefit of anchoring for generalisation of learned meanings to new contexts.

We should also note that, in our analyses, the diversity benefit for new sentences was also non-significant for the non-anchoring experiment (Norman et al., 2022). Given that these analyses were conducted on the same data, the discrepancy must be due to the model structure. Whereas Norman et al.’s model included random intercepts for participants and items and a random slope for contextual diversity by participants, ours had no random intercepts but included random slopes for contextual diversity for both participants and items. It could therefore be that the benefit of diversity is restricted to certain items and including a by-items random slope in the current analyses accounted for some of this variance, rendering the diversity benefit non-significant. This requires exploration in future studies, but the fact that the significance of this effect is somewhat dependent on model structure certainly suggests that it may not be very robust.

For old sentences, accuracy was higher for low relative to high diversity items in both experiments, although the difference was numerically smaller in the current anchoring experiment. Furthermore, for these old sentences, accuracy for high diversity items was greater in the current anchoring experiment than in the non-anchoring experiment. Together these results align with the second part of our prediction, that anchoring would reduce the benefit of the low relative to the high diversity condition for sentences drawn from a familiar context. Overall, anchoring boosted performance for high diversity items in the familiar contexts, presumably because participants had received five rather than just one exposure to this context during training. However, anchoring did not boost performance for these high diversity items in new contexts that required generalisation of meaning knowledge.

### Form learning

The results of the current experiment replicate Norman et al. (2022), who obtained no difference in performance in an old-new decision task for new words learned in high versus low diversity sentence contexts. This is consistent with some other studies that have also found no effect of contextual diversity on word form learning (Bolger et al., 2008; Hulme et al., 2023). However, the findings are in contrast to Johns et al. (2016), who found a benefit of contextual diversity during new word learning for both accuracy and RTs in an old-new decision task. It is also contrary to Mak et al. (2021), who showed that including an anchoring phase, which familiarised participants with one context before diversity was introduced, induced a benefit of contextual diversity on old-new decision RTs. As suggested by Norman et al., one reason that ours and these previous findings differ could be the type of foils used. Our foils were pseudowords that deviated from the target words by one letter, whereas Johns et al. (2016) and Mak et al. (2021) used untrained pseudowords that were constructed in the same way as the target words but were far less similar. Accordingly, the task was more challenging for participants in our experiment, as evidenced by longer RTs (~1100 ms in our experiment, vs. ~650–700 ms in John’s et al. and Mak et al.). This may indicate that our (and Norman et al.’s) participants used a different response strategy that involved focusing on the precise orthographic form of the words, rather than making a judgement based on overall familiarity. This may have, in turn, reduced the influence of semantics in our task and thus eliminated any influence of diversity. This explanation is, of course, speculative and requires further investigation.

#### Meaning learning

The cloze task differs from tasks more commonly used to assess semantic knowledge in that it explicitly tests both use of the learned meaning in a familiar context and generalisation of the learned meaning to new contexts. For the familiar contexts, we replicated Norman et al. (2022) in that performance was better for low relative to high diversity items. This aligns with previous experiments that have found a low diversity benefit on semantic relatedness judgements (Hoffman & Woollams, 2015; Johns et al., 2016; Mak et al., 2021 Experiment 1) and recall of semantic features of new meanings (Hulme et al., 2023). As outlined in the “Introduction” section, based on simulations of spreading activation in lexical networks, Mak et al. argued that low diversity helps to associate new words with prior lexical knowledge by creating a dense immediate semantic neighbourhood. This is a good account of the low diversity benefit for familiar context cloze sentences for which good performance required a strong association with one context. It is also in line with the fact that the low diversity benefit was reduced in the current experiment relative to that seen in Norman et al., since the inclusion of an anchoring phase increased the association with the familiar context for high diversity items.

For new contexts, unlike in Norman et al. (2022), performance in the current experiment was not significantly better for high than low diversity items (though numerically, the effect was in the same direction). This was unexpected since, based on Mak et al.’s (2021) finding that including an anchoring phase eliminated the semantic relatedness judgement benefit for low diversity items, we expected anchoring to benefit rather than hinder performance for high diversity items in the cloze task. However, neither Mak et al. nor other previous studies explicitly tested generalisation of learned meanings to new contexts, therefore, the only appropriate comparison is with Norman et al. In the current study, the introduction of anchoring necessitated a reduction in the number of contexts experienced in the high diversity condition relative to Norman et al. (6 vs. 10 contexts, respectively) to match the total number of exposures between conditions (10 in both experiments). Our results therefore suggest that more diversity may be better if the intended outcome is the ability to generalise learned meanings to new contexts. Relating this to the simulations of Mak et al., the high diversity condition in the current anchoring experiment may not have sufficiently increased the number of unique word nodes with which the diverse words were associated. Further research with more naturalistic training regimes (e.g., over days or weeks) should explore the relative importance of securely associating a new word with existing lexical knowledge versus experiencing it in many different contexts for both word form and meaning learning, and in different semantic tasks.

#### Limitations and future research

One key issue for future research is how many contexts are necessary to see advantages for high relative to low diversity exposure. With respect to form learning, number of contexts does not seem to have a systematic effect. Mak et al. (2021) and Johns et al. (2016) obtained a benefit of high diversity in old-new decision with two and six contexts respectively, whereas our study and Norman et al.’s (2022) saw no effect and included six and ten contexts. Thus, other factors may be more important with respect to whether a diversity advantage is observed in word recognition tasks, such as the extent to which the task relies on overall familiarity vs. precise orthographic judgements and, relatedly, the extent to which semantic knowledge is drawn on in the task. Regarding meaning learning, fewer contexts seems to be best if the task does not require generalisation. Johns et al. and Mak et al. (Experiment 1) obtained a low diversity benefit in synonym judgement tasks using five and six contexts in the high diversity condition respectively, but this was eliminated in Mak et al. (Experiment 2) with just two contexts in the high diversity condition, though this could also be due to the anchoring phase boosting performance in the high diversity condition. In our work, considering just the old cloze sentences that did not require generalisation, Norman et al. observed a low diversity benefit with ten contexts in the high diversity condition, but this benefit was reduced in the current experiment which had six contexts in the high diversity condition. Thus, when the task requires using a word in a familiar context, the more additional contexts the word has been experienced in, the poorer performance seems to be, though again this could be a trade-off with relatively less experience in the familiar context, that is, the ratio between the two. However, when generalisation is necessary the opposite seems to be true. Norman et al. obtained a diversity advantage for new cloze sentences with ten contexts in the high diversity condition, whereas in the current experiment, this benefit was no longer significant with six contexts in the high diversity condition. Thus, more versus fewer contexts seem to have opposing benefits for using a word in familiar versus new environments.

A second issue that warrants discussion is how anchoring, and indeed low diversity, are operationalised. In the current experiment, the low diversity condition consisted of 10 sentences on the same relatively broad topic (e.g., animals, law, or weather), and the anchoring phase used 5 of these sentences. This is somewhat different from Mak et al. (2021), in which topics were more specific (e.g., Donald Trump, David Bowie, or Brexit) and anchoring therefore linked a new word to one specific topic. Anchoring could perhaps be more beneficial in the latter situation since participants may find it easier to form associations between target word meanings and specific topics. However, the fact that we found that anchoring improved cloze task performance in the high diversity condition for familiar context sentences, does suggest that participants were sensitive to the association between word meanings and our broad topics. Furthermore, the approach taken in the current experiment more closely mimics the distinction between high- and low-diversity words seen in natural language. Nevertheless, future experiments should explore the optimal breadth of topics in both initial and subsequent exposures for supporting word form and meaning learning.

A third consideration for future work is the relationship between contextual diversity and semantic ambiguity. Joseph and Nation (2018) primarily manipulated topic, with adults rating the high diversity sentences as more similar in topic than the low diversity sentences. Though not explicitly stated, the degree to which the meaning conveyed by the target word varied across sentences was not intended to differ between diversity conditions. Our exploratory analysis, in which an additional group of participants typed in a word that best fit the blank in each of the training sentences, provides quantitative evidence to support this suggestion. The responses given for the high diversity sentences were slightly more, rather than less, similar to the underlying target word meaning than those given for the low diversity sentences, and were no more variable. This suggests that meaning was not harder to predict nor more variable in the high than the low diversity condition in the current study. Several authors have used latent semantic analysis (LSA; Bolger et al., 2008; Hoffman & Woollams, 2015; Hsiao et al., 2020; Mak et al., 2021) or a related metric of semantic distinctiveness (Johns et al., 2012, 2016) to measure/manipulate contextual diversity. These metrics assess the overlap between the words used in the different text contexts in which a word occurs. Although LSA has been suggested to capture semantic ambiguity (Hoffman et al., 2013), Cevoli et al. (2021) argued that this was not the case, but rather that it reflects the “topics and types of written material in which words occur” (p247). Thus, the majority of work on contextual diversity may be better thought of as manipulating topic rather than semantic ambiguity. However, more work is clearly needed to disentangle the contribution of these factors to both lexical and semantic processing tasks (Hulme et al., 2023).

Some final issues to consider for word learning studies are how similar tasks are to natural language learning and processing. In exploratory analyses, we observed moderate correlations between English lexical knowledge (i.e., performance on the ROAR and LexTALE) and the tasks we used to assess word learning (i.e., old-new decision and cloze). This suggests that our study did tap into participants’ natural language processing skills, rather than, for example, targeting more general problem-solving skills. However, our experimental tasks were not optimised to measure individual differences (see Blott et al., 2023; Goodhew & Edwards, 2019), and future research should further explore the strategies that participants use when engaging in word-learning experiments. Another difference from encountering new words in natural reading situations is that we explicitly instructed participants to learn the form and meaning of new words. Future work could adopt an incidental learning design where participants encounter new words through reading stories and are not aware they will be tested (e.g., Hulme et al., 2019; Hulme & Rodd, 2021). This may change the advantages of low versus high diversity as well as anchoring. We also acknowledge that, by replacing real (known) words with pseudowords, our experiment perhaps primarily examines form-to-meaning learning, rather than meaning learning itself. This decision was largely made for pragmatic reasons. Even typical adults reading in their native language find it difficult to learn several new words in a single session (as demonstrated by the far from ceiling performance on the cloze task), and this is exacerbated if the to-be-learned words are also new concepts. However, it will be important for future studies to examine new form and concept learning, since this is more akin to the situation people face when encountering new words in text in their native language in everyday life. Finally, although our approach provided control over confounding variables such as prior word knowledge and the informativeness of sentence contexts, we only examined learning within a single session, whereas natural word learning occurs over a protracted period with encounters spaced out over time (Pagán & Nation, 2019). This opportunity for consolidation would likely change the relative benefits of high and low contextual diversity as well as how anchoring processes might operate.

## Conclusion

This study examined whether including an anchoring phase before introducing contextual diversity changes the relative benefits of learning new words in high versus low diversity sentence contexts for form and meaning learning. As in Norman et al.’s (2022) previous study that used the same learning paradigm and stimuli but did not include an anchoring phase, we observed no effect of contextual diversity on old-new decision accuracy or RTs. Thus, anchoring did not induce a benefit for high-diversity items in the current paradigm. It may be that using foils that are less similar to the target words (e.g., Johns et al., 2016; Mak et al., 2021) is necessary to observe semantic effects in this task, since using similar foils forces participants to pay close attention to orthographic form. With regards to meaning learning, for cloze sentences that required generalisation to a new context, performance was non-significantly higher for high than low diversity items, whereas for those that used a familiar context, performance was better for low diversity items. Comparing our results to those of Norman et al. suggested that while anchoring did serve to secure new words to a particular context, it did not help generalisation to new contexts. Future research should aim to disentangle how both initial exposure to one context and subsequent exposure to a varying number of contexts benefit word form learning and generalisation of meaning knowledge.

## Supplemental Material

sj-docx-1-qjp-10.1177_17470218231218990 – Supplemental material for Contextual diversity and anchoring: Null effects on learning word forms and opposing effects on learning word meanings Supplemental material, sj-docx-1-qjp-10.1177_17470218231218990 for Contextual diversity and anchoring: Null effects on learning word forms and opposing effects on learning word meanings by Jiayin Li, Louise Wong, Catarina Rodrigues, Rachael C Hulme, Holly Joseph, Fiona E Kyle and J S H Taylor in Quarterly Journal of Experimental Psychology
