# Supplementary material for: Contextual diversity and anchoring: Null effects on learning word forms and opposing effects on learning word meanings
Source: Q J Exp Psychol (Hove). 2024 Sep 17;77(11):2180–98. doi: 10.1177/17470218231218990 (PMC11528881; doi:10.1177/17470218231218990)
Supplement: sj-docx-1-qjp-10.1177_17470218231218990 – Supplemental material for Contextual diversity and anchoring: Null effects on learning word forms and opposing effects on learning word meanings [file sj-docx-1-qjp-10.1177_17470218231218990.docx]

**Contextual diversity and anchoring: Null effects on learning word forms and opposing effects on learning word meanings**

Jiayin Li^1,2^*, Louise Wong^2^*, Catarina Rodrigues^2^, Rachael C. Hulme^2,3^, Holly Joseph^4^, Fiona E. Kyle^2^, J. S. H. Taylor^2^.

1. School of Psychology and Clinical Language Sciences, University of Reading

2. Division of Psychology and Language Sciences, University College London

3. Department of Psychology and Centre for Applied Behavioural Sciences, Heriot-Watt University

4. Institute of Education, University of Reading

*joint first authors

Open Science Statement: This project was pre-registered on AsPredicted.org (https://aspredicted.org/4ZD_N4R). All data, scripts and experimental materials are publicly available on Open Science Framework (<https://osf.io/rx6t4/>). The main experimental tasks are also available on Gorilla Open Materials (<https://app.gorilla.sc/openmaterials/612824>)

**Appendix A**

Supplementary Table 1. Demographic details of included participants (N = 278)

|  | Number of participants |
| --- | --- |
| **Gender** |  |
| Female | 173 |
| Male | 100 |
| Non-binary | 5 |
| **Education level, years of education** |  |
| GCSE (or equivalent), 10yrs | 28 |
| A-Level (or equivalent), 12yrs | 67 |
| Technical/Vocational/Associate’s degree, 14yrs | 10 |
| Undergraduate degree, 15yrs | 124 |
| Master’s degree, 16yrs | 42 |
| Doctorate degree, 20yrs | 7 |
| **Language background** |  |
| Monolingual | 230 |
| Bilingual/multilingual | 48 |

**Appendix B**

Supplementary Table 2. Sentence stimuli used in the learning phase for all eight target words in the low diversity (LD) and the high diversity (HD) conditions.

| Sentence Stimuli | Diversity | Learning Phase | |
| --- | --- | --- | --- |
| **accumulated** | | | |
| Enough proof had ________ so that the jury could make a fair judgement on the case. | Low | anchoring |  |
| The police ________ a lot of strong evidence which meant they could arrest the thief. | Low |  |  |
| Members of MI5 ________ all the incoming data and saved it onto a computer file. | Low |  |  |
| After the news report went out, the police ________ more than 25 witnesses. | Low |  |  |
| The lawyer ________ witness statements to get support for the case. | Low |  |  |
| The burglar ________ information about the neighbourhood before committing the crime. | Low | post-anchoring |  |
| The evidence ________ until there was no question that he was guilty. | Low |  |  |
| The proof that she had stolen the money ________ over time and eventually she lost her job. | Low |  |  |
| The witness statements ________ and in the end he decided to plead guilty. | Low |  |  |
| The solicitor ________ the documents for the case and took them to court. | Low |  |  |
| Lava had ________ beneath the surface which caused a spectacular eruption from the volcano. | High |  |  |
| His debts ________ until he had to sell his house to pay off the loan. | High |  |  |
| Although she had ________ a lot of wealth, this meant she also had to pay a lot of tax. | High |  |  |
| She was shocked to discover how many emails had ________ while she was away. | High |  |  |
| The fluid had ________ in his lungs and he found it very hard to breathe. | High |  |  |
| **Amalgamate** | | | |
| The British and Americans ________ their designs for a new fighter plane. | Low | anchoring |  |
| Due to Government cut backs the two regiments ________ with each other. | Low |  |  |
| The army ________ plans with the local charity in order to provide medical care. | Low |  |  |
| If the two countries stopped fighting and ________ into one nation then the war would end. | Low |  |  |
| The goggles and helmets were ________ so that there was less kit to carry. | Low |  |  |
| Nuclear submarines are powered by ________ heat and water so they don't need to refuel. | Low | post-anchoring |  |
| The Generals ________ their knowledge of warfare to help the Queen beat the enemy. | Low |  |  |
| Army scientists have ________ many materials to make bomb proof jackets. | Low |  |  |
| The generals ________ their ideas for removing their equipment from Afghanistan. | Low |  |  |
| The navy ________ designs for airports and ships to create aircraft carriers. | Low |  |  |
| The director ________ a traditional play with pop music to create a brand new musical. | High |  |  |
| The two universities ________ last year and now there are 15,000 students in total. | High |  |  |
| When the Spanish invaded Mexico, the two cultures ________ quite well. | High |  |  |
| The scientific findings were ________ in order to find a cure for the disease. | High |  |  |
| The two companies ________ and hoped that they would make twice as much money. | High |  |  |
| **Intervened** | | | |
| The President ________ swiftly in the civil war and most of the people were grateful. | Low | anchoring |  |
| The government ________ during the teachers' strike and gave them more money. | Low |  |  |
| They discussed whether it would be acceptable if the US ________ in the Syrian crisis. | Low |  |  |
| The UK has ________ in other countries' actions when there are human rights abuses. | Low |  |  |
| The government ________when the economy collapsed to make sure the banks could function. | Low |  |  |
| The transport secretary ________ to say that all pensioners should get free travel. | Low | post-anchoring |  |
| The president ________ before the execution and saved the prisoner's life. | Low |  |  |
| The health secretary ________ and approved the new cancer treatment. | Low |  |  |
| The Council should have ________ much earlier to stop the riots, but they didn't. | Low |  |  |
| The government ________ and as a result, prevented the bill from being passed. | Low |  |  |
| Social Services ________ to help the young people have their meetings in the community hall. | High |  |  |
| The farmer had ________ months earlier so that all the locals could make use of the track. | High |  |  |
| One brave girl ________ and managed to prevent the playground fight becoming more serious. | High |  |  |
| Sally hadn't ________ when she witnessed the bullying but she did tell a teacher. | High |  |  |
| The mother ________ in the early stages of her children's fights, for the sake of peace. | High |  |  |
| **Exacerbated** | | | |
| Cigarette smoking ________ the man's breathing difficulties, but he just couldn't stop. | Low | anchoring |  |
| His infection was ________ because the hospital didn't have any antibiotics to give him. | Low |  |  |
| The heat had ________ the swelling in her broken arm, so the plaster became too tight. | Low |  |  |
| The pain in her leg ________ her bad mood, and she ended up taking more pain killers. | Low |  |  |
| Doctors not washing their hands properly may have ________ the spread of the disease. | Low |  |  |
| A study showed that not using waterproof sun cream ________ levels of sunburn. | Low | post-anchoring |  |
| Eating greasy food ________ his weight problems, which caused him a great deal of upset. | Low |  |  |
| Sitting in a dusty room ________ her daughter's asthma, so they asked to sit outside. | Low |  |  |
| Eating cake ________ Sally's diabetes, so next time she'll go for a healthier option. | Low |  |  |
| My granny said that going to bed with wet hair had ________ my cough and made me even more ill. | Low |  |  |
| Watching the film 'Madagascar' ________ Louise's longing to go on holiday somewhere hot. | High |  |  |
| Growing up in a busy city ________ Jake's hatred of noise so he moved to the country. | High |  |  |
| She tried to calm him down but she just ________ the situation and he became very angry. | High |  |  |
| The horrible sound of an aeroplane overhead was ________ by my dad playing loud music. | High |  |  |
| The death of Melissa's dog ________ her unhappiness, so her mother bought her a new puppy. | High |  |  |
| **Confabulated** | | | |
| The Year 6 football team ________ about their past victory on the way to the match. | Low | anchoring |  |
| The school children ________ in a made-up language so no-one else could understand them. | Low |  |  |
| Jessica loved the way that she and her mates ________ with children in school. | Low |  |  |
| If children ________ in class and the teacher heard them, she got very cross. | Low |  |  |
| The head teacher ________ with the other teachers whilst eating his lunch. | Low |  |  |
| The teachers were all very friendly and often ________ with the children after school. | Low | post-anchoring |  |
| The new girl ________ cheerfully with everyone, and soon made lots of friends at school. | Low |  |  |
| The mums ________ for so long in the playground that the teacher asked them to leave. | Low |  |  |
| The dinner ladies ________ cheerily with the children as they served them their meals. | Low |  |  |
| The students ________ about why Johnny had been sent to the head teacher's office. | Low |  |  |
| He ________ in such a loud manner, that I had to move to the back of the room. | High |  |  |
| I wish I had ________ more with my granny when I was young: she had some amazing stories. | High |  |  |
| The children met on holiday and ________ happily although they spoke only a little English. | High |  |  |
| After the match, the hockey team ________ about what had gone well and what had gone badly. | High |  |  |
| The journalist was exhausted as the famous actor ________ for four hours without a break. | High |  |  |
| **Languished** | | | |
| The animals had ________ because they were weren't being looked after properly. | Low | anchoring |  |
| The dog ________ in her basket for a week, before her owner realised she was pregnant. | Low |  |  |
| The farmer claimed that the horse ________ after her foal was taken away from her. | Low |  |  |
| When the old lady became unwell, her canary ________ as there was nobody to feed him. | Low |  |  |
| The battery hens on the farm ________ as their living conditions were very poor. | Low |  |  |
| My cat ________ for days, so I gave her a new brand of cat food and she perked up. | Low | post-anchoring |  |
| The firefighters think the animals ________ because it was difficult for them to breathe. | Low |  |  |
| Protesters released the pigs, as they had ________ in filthy conditions for too long. | Low |  |  |
| The hamster had ________ for several days, and the vet recommended a vitamin injection. | Low |  |  |
| The crocodile ________ sadly by the river, mourning her baby who had died. | Low |  |  |
| The child ________ in hospital until the doctors changed her medicine which helped a lot. | High |  |  |
| The soldiers ________ for so long, that they were not fit to fight when the battle started. | High |  |  |
| I'm an awful gardener: my cabbages ________ for a week before I realised they needed watering. | High |  |  |
| The prisoners ________ for days on end in their cells before receiving medical attention. | High |  |  |
| The plants had ________ and eventually died, because the sprinkler system failed. | High |  |  |
| **Thwarted** | | | |
| Their progress was ________ by a  fallen tree. | Low | anchoring |  |
| The search was ________ by the heavy flooding. | Low |  |  |
| The family walk was ________ by a sudden thunder storm. | Low |  |  |
| The explorers’ climb of Everest was ________ by an avalanche. | Low |  |  |
| The marathon runners were ________ by temperatures over 40 degrees. | Low |  |  |
| Our holiday plans were ________ by a tsunami in southern Thailand. | Low | post-anchoring |  |
| Our attempts to cross the river were ________ by the strong currents. | Low |  |  |
| His plan to go bungee jumping was ________ by high winds. | Low |  |  |
| Their plans to play outdoors were ________ by the sudden hail storm. | Low |  |  |
| They tried to go sledging but were ________ by the melting snow. | Low |  |  |
| His attempt to kill the Prime Minister was ________ by a nearby guard. | High |  |  |
| The cricketer’s promising career was ________ by serious injury. | High |  |  |
| A terrorist attack was ________ by the soldiers who found the bomb before it exploded. | High |  |  |
| So far his plan to grow vegetables in the garden has been________ by a lack of seeds. | High |  |  |
| The robbery was ________ by the police who managed to enter the building and arrest the raiders. | High |  |  |
| **Divulged** | | | |
| The judge ________ all of the criminal’s previous offences to the shocked jury who had found him not guilty. | Low | anchoring |  |
| The CCTV video ________ the footage needed to arrest the suspect. | Low |  |  |
| The lawyer was very thorough and  ________ all of the details of the case to the judge. | Low |  |  |
| After witnessing a crime, she ________ everything she saw to the police. | Low |  |  |
| The suspect ________ no details of where he was on the night of the crime. | Low |  |  |
| The solicitor wanted more information but it could not be  ________ as it was not relevant to the case. | Low | post-anchoring |  |
| Under questioning, the witness ________ what he remembered but it wasn't enough to convict the burglar. | Low |  |  |
| The police officer ________  the details of the court case to the solicitor. | Low |  |  |
| The young man in the dock ________ the identity of the woman who had attacked him. | Low |  |  |
| In an unexpected turn of events, the judge  ________ where the documents were being held. | Low |  |  |
| When the magician refused to pay her more, his assistant ________ all his secret tricks. | High |  |  |
| His ex-girlfriend ________ many of the details of their relationship, much to his embarrassment. | High |  |  |
| The newspaper headline ________ top secret information obtained from MI5. | High |  |  |
| After being held prisoner for three days, the suspected terrorist ________ his password details. | High |  |  |
| The government never ________ any personal data as this was against the law. | High |  |  |

**Appendix C**

Supplementary Table 3. Sentence stimuli used in the cloze task.

| Sentence Stimuli | Target word meaning | Sentence Type |
| --- | --- | --- |
| The police ________ evidence against the suspect at a very rapid pace. | Accumulated | old |
| Pondweed ________ in the garden pond as it hadn’t been cleaned for months. | Accumulated | new |
| The head of the army found it helpful when he ________ his invasion plans with those of the SAS | Amalgamated | old |
| Two cultures can be ________ quite easily if the same language is spoken. | Amalgamated | new |
| The teachers ________ so loudly that the students had to ask them to be quiet. | Confabulated | old |
| My parents were angry because I ________ on the phone too long and the bill was huge. | Confabulated | new |
| After hours of questioning, the suspect ________ the location of the stolen jewels. | Divulged | old |
| The CEO ________ the details of the company’s losses to the board to try and save the business. | Divulged | new |
| The lack of clean bandages ________ the chances of the patient’s cut becoming infected. | Exacerbated | old |
| The sisters’ bad moods ________ the chances of arguments during the holiday. | Exacerbated | new |
| The government only ________ in other countries’ problems in order to save lives. | Intervened | old |
| She was worried that if she ________ in the argument someone would turn on her. | Intervened | new |
| The cow had ________ for too long, and the vet was worried that she may die. | Languished | old |
| After the sudden death of her husband, the woman ________ in grief for many years. | Languished | new |
| They wanted to trek across the desert, but were ________ by a sudden sandstorm. | Thwarted | old |
| The prince’s rescue attempt was ________ by the fierce dragon | Thwarted | new |

**Appendix D**

Supplementary Table 4. List of instructions presented during the learning and testing phases.

| **Task** | **Instructions** |
| --- | --- |
| **Learning phase** | In the first task you will read a series of short sentences describing the meanings of new words. Please read these carefully (silently, in your head) and try to learn the new words and what they mean from the information provided. **You will be tested on the new words and their meanings later in the study**.  You'll need to read each sentence carefully to understand the full meaning of the new words.  After some of the sentences you will be asked questions to check your understanding.  Please **do not** write anything down, just read the passages carefully and try to learn the new words and their meanings.  You will have the option to take breaks at set times during the task. Please try and stay focused on the task in between these scheduled breaks and complete it **in one go**.  Click "Next" when you are ready to begin reading the sentences.  This task will take you approximately 15 minutes. |
| **Old-new decision task** | In this task, you will be presented a series of words. Some will be the same as those you learned in the previous task, others will be spelled incorrectly.  Your task is to judge **as quickly and as accurately as possible** whether the word on screen is one you learned in the previous task.  On your keyboard, place your left index finger on the 'F' key and your right index finger on the 'J' key.  **When the word is spelt correctly, press the 'F' key.**  **When the word is spelt incorrectly, press the 'J' key'.**  Press the space bar on your keyboard to begin the task. |
| **Cloze task** | In this task you will see a series of new sentences, one at a time. Each of these sentences has a word missing, which will be one of the new words you learned earlier. Your task is **to complete the sentences by selecting the correct word.**  Please read each sentence carefully before clicking on the new word that correctly completes it.  There is more than one sentence that goes with each word.  There are two practice questions at the beginning after which you will receive feedback, but there is no feedback in the main part of  the task. |

**Appendix E**

Analysis of contextual constraint of sentences

A linear mixed effect model was constructed to examine the difference in similarity scores (i.e. the similarity between a participant’s response and the target word) between high diversity and low diversity sentences. Similarity score, as the outcome variable, was coded as ordinal data due to the restricted range of scores that were possible. A cumulative link model was fitted using the package ordinal (Christensen, 2019). The final model was the simplest model and contained only the fixed effect of diversity (high vs. low) and by-item and by-participant random intercepts. As shown in Supplementary Figure 1, similarity scores for high diversity sentences (*M* = 0.67, *SD* = 0.20) were significantly higher (*χ^2^*(1) = 8.39, *p* = .004) than those for low diversity sentences (*M* = 0.64, *SD* = 0.21).


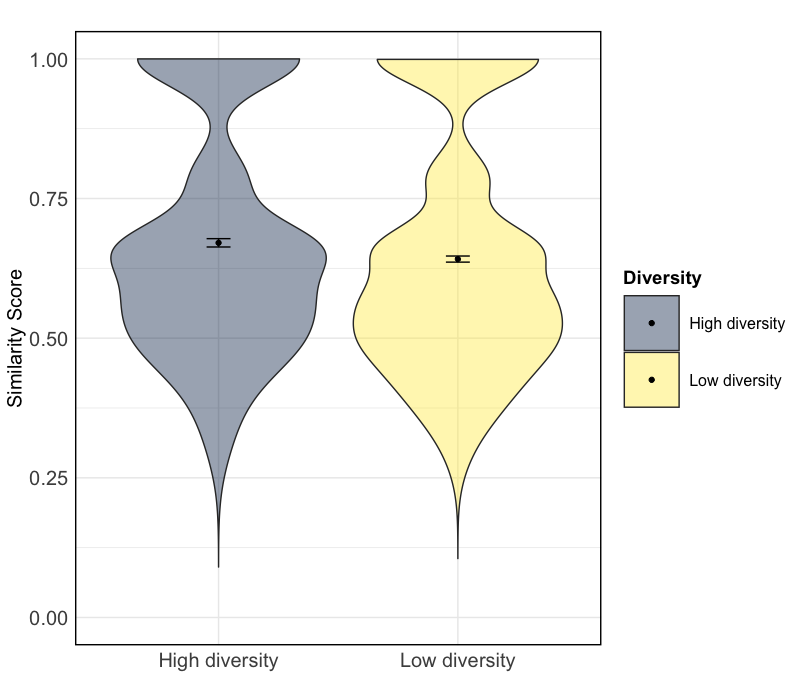


Supplementary Figure 1. Distribution of means for similarity score in high and low diversity sentences. The violin shows the distribution of the data, and the inside point (along with error bar) gives the mean scores across participants in that condition.

To examine response variability, we calculated the number of different response words for each item in the high and low diversity condition and then divided it by the total number of trials for that item, which differed between high and low diversity conditions (trial number: *N_high diversity_* = 750, *N_low diversity_* = 1477). Supplemental Figure 2 shows these proportions for each target word. A linear mixed effects model was constructed to analyse the effect of diversity on the proportion of different responses. The final model included the fixed effect of diversity (high vs. low) and a by-item random slope for diversity. The results revealed that there was no significant difference in the variability of the response words between the high and low diversity sentences (*χ^2^*(3) = 3.36, *p* = .068. *M_high diversity_* = 32%, *M_low diversity_* = 28%.


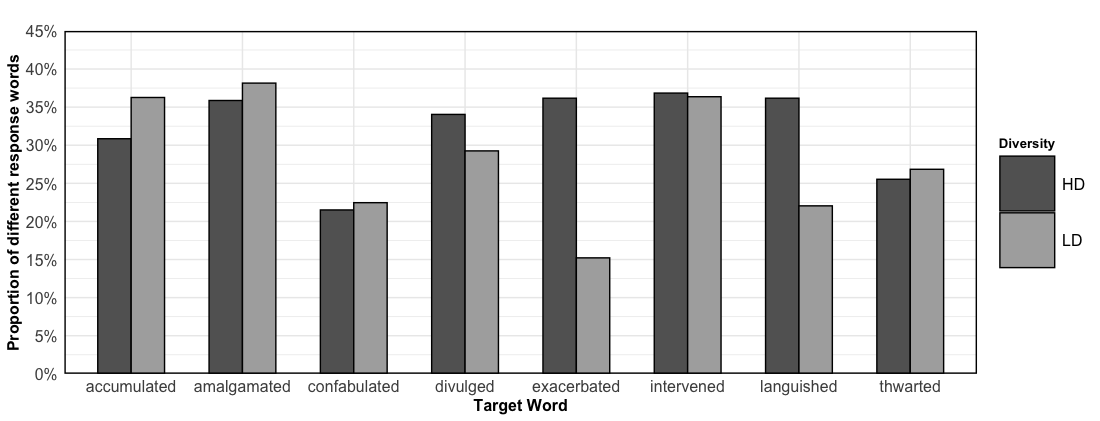


Supplementary Figure 2. The proportion of different response words for each item in the high and low diversity condition.
